# Unravelling the Puzzle: Highlighting a Case of VEXAS (Vacuoles, E1 Enzyme, X-linked, Autoinflammatory, Somatic) Mimicker Presented With Inflammatory Symptoms and Pancytopenia

**DOI:** 10.7759/cureus.94713

**Published:** 2025-10-16

**Authors:** Anurag Singh, Alka Yadav, Ankita Singh

**Affiliations:** 1 Pathology, Sanjay Gandhi Postgraduate Institute of Medical Sciences, Lucknow, IND

**Keywords:** cytoplasmic vacuolization, megaloblastic anemia, pancytopenia, uba1 gene mutation, vexas syndrome

## Abstract

VEXAS (vacuoles, E1 enzyme, X-linked, autoinflammatory, somatic) syndrome is an autoinflammatory illness that arises due to inactivating mutations in the X-linked *UBA1* gene and progresses to impaired ubiquitination, accumulation of misfolded proteins, and activation of numerous inflammatory pathways. Several reactive conditions may mimic the clinical and morphological findings of VEXAS syndrome; therefore, careful differentiation from more common diseases is essential. We present an 82-year-old female who had clinical and morphological features suggestive of VEXAS syndrome; however, a diagnosis of VEXAS syndrome was ruled out since a pathogenic *UBA1* gene mutation was not found. Further evaluation established a final diagnosis of megaloblastic anemia with masked macrocytosis, which closely simulated the hematological manifestations of VEXAS syndrome. This case study emphasizes how challenging it is to diagnose VEXAS syndrome mimickers and how crucial it is to do a systematic clinical and laboratory assessment in order to distinguish it from other common illnesses with uncommon presentations.

## Introduction

VEXAS (vacuoles, E1 enzyme, X-linked, autoinflammatory, somatic) syndrome is a newly identified disease that was initially characterized in 2020. This autoinflammatory illness results from inactivating mutations in the X-linked *UBA1 *gene, leading to impaired ubiquitination, accumulation of misfolded proteins, and activation of many inflammatory pathways [1].

VEXAS syndrome can resemble common illnesses, particularly recurrent polychondritis, vasculitis, and Sweet syndrome. Distinguishing VEXAS syndrome from these mimickers is clinically crucial, as the management strategies and prognostic outcomes differ significantly. While VEXAS usually requires immunosuppressive and anti-inflammatory therapy and may carry a progressive or even life-threatening course, many of its mimickers, such as reactive inflammatory causes, are reversible with appropriate and timely treatment. Precise distinction thereby averts unwarranted exposure to immunosuppressive drugs and ensures disease-specific therapy, enhancing patient outcomes [2].

It generally manifests as a progressive systemic inflammatory disorder in males over the age of 50 years. The most common reported clinical features include fever, skin involvement, and hematological manifestations [1,2]. The hematological changes can vary from macrocytic anemia to myelodysplastic syndromes (MDS) and plasma cell abnormalities, with many cases presenting concurrently with MDS [2,3].

A provisional diagnosis of VEXAS syndrome is frequently considered among clinicians due to a combination of the unusual constellation of symptoms and signs. The management of this hematoinflammatory illness typically necessitates substantial doses of corticosteroids, with various steroid-sparing therapies, interleukin-1 inhibitors, and allogeneic hematopoietic stem cell transplantation [2-4]. Here, we report a challenging case of vitamin B12/folate deficiency-induced masked macrocytosis and pancytopenia, cytoplasmic vacuolization in erythroid and myeloid lineage cells, and systemic inflammatory symptoms that closely resembled VEXAS syndrome.

## Case presentation

**Figure 1:**
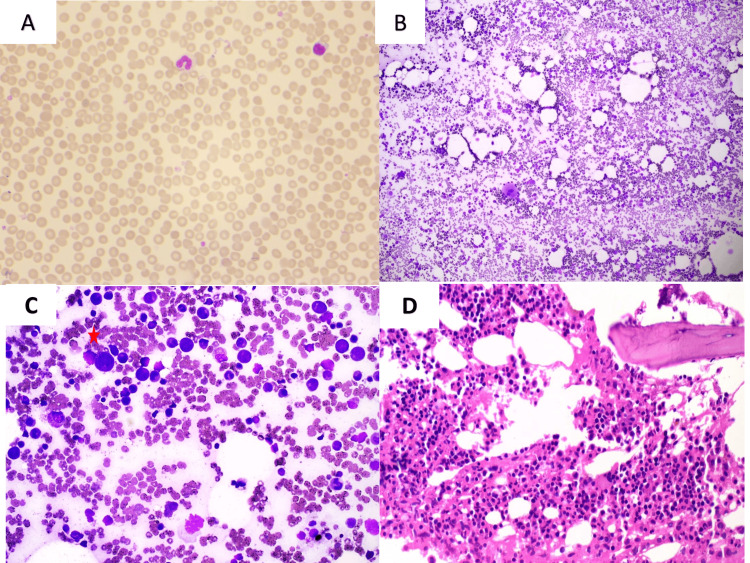
(a) Peripheral blood smear showed normocytic normochromic red blood cells and pancytopenia (Leishman stain, 40×). (b) Bone marrow aspirate smear was hemodiluted and displayed trilineage hematopoiesis (Leishman stain, ×10). (c) Bone marrow aspirate smear displayed cytoplasmic vacuolization* in myeloid and erythroid lineage cells. (d) Bone marrow biopsy showed trilineage hematopoiesis and normoblastic erythroid hyperplasia.

An 82-year-old woman with fever, fatigue, recurring rash, and symmetric polyarthritis of small and large joints. No cough, malaria exposure, or tuberculosis contact was reported. Her physical exam revealed no lymphadenopathy, clubbing, or eschar. Neurological and respiratory findings were normal. Her chest X-ray and abdomen ultrasound were normal. Her blood test results showed low hemoglobin (9.8 g/dL), a total leucocyte count (TLC) of 3.0 × 10³/µL, an absolute neutrophil count of 1.6 × 10³/µL, and a platelet count of 75 × 10³/µL. The peripheral blood smear showed normocytic and normochromic red blood cells (Figure 1a). Mean corpuscular volume (MCV) was 98.7 fL, approaching the macrocytic range. Her liver function (total bilirubin: 0.90 mg/dL; direct bilirubin: 0.30 mg/dL) values were within normal limits, indicating no evidence of hepatic dysfunction. Her serum lactate dehydrogenase (LDH) was 540 U/L (reference: 85-450 U/L), mildly raised. Thus, pancytopenia was initially linked to systemic inflammatory disorders, but rheumatoid arthritis (RA) and systemic lupus erythematosus (SLE) lab tests were negative. Laboratory tests revealed elevated C-reactive protein (83.0 mg/L), serum ferritin (961 µg/L), and anti-cyclic citrullinated peptide (anti-CCP) levels of >300.0 units. The serum iron was normal. The patient was initiated on systemic prednisolone and methotrexate for autoimmune inflammatory illness. Even after both medicines, her inflammation persisted. Prednisolone was partially tapered, and infliximab was given as anti-TNF-α therapy. The patient developed polychondritis and urged further assessment for an autoinflammatory condition, raising suspicion for a rare autoimmune inflammatory disease. Anakinra, an IL-1 receptor antagonist, was given but stopped after 7 days due to severe injection site reactions. She got partial symptomatic relief, but pancytopenia persisted. The initial diagnostic work-up focused on ruling out autoinflammatory disorders due to complex clinical symptoms with prolonged pancytopenia, systemic inflammatory signs, and partial response to immunosuppressive medication. No definitive diagnosis was made. A provisional diagnosis of VEXAS syndrome was suggested, and a bone marrow examination was advised. In bone marrow aspirate smears, trilineage hematopoiesis with modest erythroid hyperplasia and megaloblastic changes was seen. A few myeloid and erythroid lineage cells show cytoplasmic vacuolization; however, dyserythropoiesis was not significant. Megakaryocytes were adequate in number and showed normal morphology. Plasma cells and lymphocytes were within normal limits. There were no blasts seen (Figures 1b-1d).

There were no ring sideroblasts on Perl stain, and significant marrow iron stores (+2) were present. UBA1 gene mutation testing was performed in this case utilizing a Next-Generation Sequencing (NGS)-based targeted gene panel containing the hotspot area (including exon 3 at methionine-41), chosen for its high sensitivity in finding somatic variants; however, it was negative. A subtle bone marrow finding of mild erythroid hyperplasia with transitional megaloblastic changes raised suspicion for a possible megaloblastic anemia.

Serum values of vitamin B12 (100 pg/mL; reference 180-900) and folate (3.9 ng/mL; reference 4-24) were evaluated, which, in conjunction with bone marrow findings, supported the diagnosis of megaloblastic anemia with masking macrocytosis. We began her on injectable vitamin B12 (1000 µg weekly for 6 weeks), then monthly maintenance and oral folic acid (5 mg/day) were administered.

After three months, vitamin B12 (650 pg/mL) and folate (5.9 ng/mL) levels reverted to normal, and hematological parameters improved (hemoglobin 11.0 g/dL, TLC 4.2 × 10³/µL, and platelet count 160 × 10³/µL). Her inflammatory symptoms and weakness also subsided in substantial follow-up (Table 1).

**Table 1:** Hematological, biochemical parameters, and clinical symptoms before and after treatment.

Parameter/Symptom	Before Treatment	After Treatment (3 Months)
Hemoglobin (g/dL) (Ref: 12–15)	9.8	11.0
Total Leukocyte Count (×10³/µL) (Ref: 4–11)	3.0	4.2
Platelet Count (×10³/µL) (Ref: 150–450)	75	160
Mean Corpuscular Volume (MCV) (fL) (Ref: 80–100)	98.7 (near-macrocytic range)	92.1
Serum Vitamin B12 (pg/mL) (Ref: 180–900)	100	650
Serum Folate (ng/mL) (Ref: 4–24)	3.9	5.9
Symptoms	Fever, fatigue, recurring rash, symmetric polyarthritis, polychondritis	Symptoms resolved; improved strength; inflammatory manifestations subsided

## Discussion

VEXAS syndrome is a rare and newly recognized entity that typically presents as an adult-onset autoinflammatory disease. It is caused by somatic mutations in the *UBA1 *gene. The presence of this mutation in hematopoietic stem and progenitor cells leads to their clonal expansion and myeloid-skewed differentiation. VEXAS syndrome has a predilection for males [5].

The hematologic manifestations are macrocytic anemia and cytopenias with an increased risk of developing myelodysplastic neoplasm (MDS) and/or plasma cell neoplasms. The key bone marrow findings are vacuoles in myeloid/erythroid precursors; normocellular to hypercellular marrow with myeloid hyperplasia, which may progress over time; and mild dyspoiesis in uni- or multilineage may be seen. The key clinical features are fever, fatigue, arthralgias, and cutaneous manifestations. The laboratory evaluation usually reveals elevated inflammatory markers along with low serum iron [6,7].

A common condition with unusual symptoms, like megaloblastic anemia, which manifests as pancytopenia and associated inflammatory symptoms in this particular case, may be challenging to identify. Only a few instances of megaloblastic anemia have been reported, with pancytopenia having non-specific inflammatory and dermatological symptoms [8-10]. The index case was not diagnosed primarily as megaloblastic anemia because there was an absence of macroovalocytes in the peripheral blood smear during the initial examination. Masked macrocytosis is not uncommon, as it affects ~10% of patients with megaloblastic anemia [11,12].

The non-specific, ambiguous clinical and hematological symptoms of VEXAS syndrome might be confused with primary hematologic illnesses or deficiency anemia, particularly megaloblastic [13]. In the present case, a bone marrow examination was also performed, which showed mild erythroid hyperplasia with megaloblastic changes along with the presence of cytoplasmic vacuolization in a few myeloid and erythroid lineage cells. Cytoplasmic vacuolation in myeloid and erythroid lineage cells, also being a non-specific finding, can be observed in various conditions, including VEXAS syndrome, MDS, copper deficiency, and in some cases of antibiotic treatment or alcohol abuse. It is also associated with certain drug toxicities and can be an artifact of sample preparation [14]. The patient in this case study revealed increased anti-CCP titers and systemic inflammatory characteristics, which are not common for isolated megaloblastic anemia. These findings may be attributed to either a concurrent autoimmune process inducing polyclonal immune activation or to a false-positive anti-CCP result observed in specific non-rheumatologic conditions, such as chronic infections. These possibilities highlight the complexity of interpreting autoimmune markers in the presence of overlapping inflammatory and hematologic abnormalities [15]. She received immunosuppressive therapy, comprising prednisolone, methotrexate, infliximab, and anakinra, before the commencement of vitamin B12 and folate supplementation. However, the significant enhancement in hematological parameters and systemic symptoms after vitamin B12 and folate supplementation suggests that megaloblastic anemia was the primary pathology; however, the initial partial response to immunosuppressive therapy suggests the potential for a transient or overlapping non-specific inflammatory process. The index case was presented with pancytopenia and inflammatory symptoms, along with the presence of cytoplasmic vacuolization in myeloid and erythroid lineage cells on bone marrow examination; hence, the clinicians suspected a diagnosis of VEXAS syndrome. However, to make a definitive diagnosis of VEXAS syndrome, the presence of the *UBA1 *gene mutation is mandatory. A cytogenetic examination was also performed in this case, as usually patients with VEXAS syndrome are male; women need specific features to develop this pathology (X monosomy, mosaicism, or skewed X-inactivation) [16]. However, no cytogenetic abnormality was noted. The index case re-emphasizes the necessity of biochemical parameters and mimickers of rare disorders by a common disease having uncommon clinical and morphological manifestations.

## Conclusions

The difficulties in diagnosing masking macrocytosis with the presence of inflammatory symptoms from VEXAS syndrome are very challenging, especially when they are associated with bone marrow morphological findings of subtle cytoplasmic vacuolization. In suspected cases of VEXAS syndrome, before bone marrow examination and *UBA1 *gene mutation study, a meticulous evaluation for biochemical parameters for nutritional deficiency, particularly megaloblastic anemia, is necessary.
